# Advances in lentil production through heterosis: Evaluating generations and breeding systems

**DOI:** 10.1371/journal.pone.0262857

**Published:** 2022-02-18

**Authors:** Gurpreet Kaur Suri, Shivraj Braich, Dianne M. Noy, Garry M. Rosewarne, Noel O. I. Cogan, Sukhjiwan Kaur

**Affiliations:** 1 Agriculture Victoria, AgriBio, The Centre for AgriBioscience, Bundoora, Victoria, Australia; 2 School of Applied Systems Biology, La Trobe University, Bundoora, Victoria, Australia; 3 Agriculture Victoria, Grains Innovation Park, Horsham; Università Politecnica delle Marche, ITALY

## Abstract

Heterosis is defined as increased performance of the F_1_ hybrid relative to its parents. In the current study, a cohort of populations and parents were created to evaluate and understand heterosis across generations (i.e., F_1_ to F_3_) in lentil, a self-pollinated annual diploid (2n = 2× = 14) crop species. Lentil plants were evaluated for heterotic traits in terms of plant height, biomass fresh weight, seed number, yield per plant and 100 grain weight. A total of 47 selected lentil genotypes were cross hybridized to generate 72 F_1_ hybrids. The F_1_ hybrids from the top five crosses exhibited between 31%–62% heterosis for seed number with reference to the better parent. The five best performing heterotic crosses were selected with a negative control for evaluation at the subsequent F_2_ generation and only the tails of the distribution taken forward to be assessed in the F_3_ generation as a sub selection. Overall, heterosis decreases across the subsequent generations for all traits studied. However, some individual genotypes were identified at the F_2_ and sub-selected F_3_ generations with higher levels of heterosis than the best F_1_ mean value (hybrid mimics). The phenotypic data for the selected F_2_ and sub selected F_3_ hybrids were analysed, and the study suggested that 100 grain weight was the biggest driver of yield followed by seed number. A genetic diversity analysis of all the F_1_ parents failed to correlate genetic distance and divergence among parents with heterotic F_1_’s. Therefore, genetic distance was not a key factor to determine heterosis in lentil. The study highlights the challenges associated with different breeding systems for heterosis (i.e., F_1_ hybrid-based breeding systems and/or via hybrid mimics) but demonstrates the potential significant gains that could be achieved in lentil productivity.

## Introduction

Lentil (*Lens culinaris* Medik.), a self-pollinated annual diploid (2n = 2× = 14) cool season grain legume is mostly used for human consumption due to its high level of protein, vitamins, and minerals [1]. Legumes such as lentils, have been identified as a superior and cheaper protein choice over beef, poultry or fish which possess higher amounts of saturated fat and cholesterol. Amongst plant-based foods, lentil contain high levels of folate as well as β-glucans making their glycemic index exceptionally low and suitable for wide health benefits [2]. The presence of phytochemicals such as phenolic acids, flavanols, saponins, phytic acid and condensed tannins makes lentils rich in antioxidant properties [3]. During the last decade lentil production has increased from 2.8 million tonnes to 6.3 million tonnes globally, with many world markets demanding higher quality grain [4]. Australia is one of the top ten lentil producing countries, specifically regarding the red lentil. The current yield gains for lentils from conventional plant breeding in Australia are 1.13% per annum that has led to yield of 1.5 t/ha in 2017 [5, 6].

Plant breeding has helped to increase the yield and quality of legumes as well as other grain and forage crops. Many grain legume breeding programs have achieved relatively high yield gains over time, however, created domestication bottlenecks through limited breeding and biased selections based on yield potential only [7, 8]. The lentil domestication has resulted in approximately 40% loss of genetic diversity leading to narrow gene pools within breeding programs and restricted genetic gain [9]. For instance, most of the registered lentil varieties in Canada are related to the first two cultivars that founded Canadian production: ‘Laird’ and ‘Eston’ [10, 11]. Furthermore, the narrow genetic base of lentil varieties has made them more susceptible to biotic and biotic stresses [12]. Exploration of genetic diversity and introgression of novel alleles from landraces as well as crop wild relatives is pivotal for producing high yielding, disease resistant and stress tolerant varieties. Genetic diversity can be conserved whilst accelerating genetic improvement in grain legume using innovative methods of crop breeding such as hybrid breeding [13].

Conventional plant breeding in a self-pollinated crop is based on a large number of phenotypic selections that are made from genetically diverse populations, to accumulate beneficial alleles in a stepwise manner in homozygous inbred lines [14]. These inbred lines are then passed through multiple evaluation trials for various biotic and abiotic stresses. Finally, the superior selected inbred lines are multiplied and delivered by the seed industry to growers. F_1_ hybrid breeding is an alternative approach where the product delivered to growers is the first filial generation of a cross that exploits cross-breeds with improved vigor over the parental genotypes and this is referred to as hybrid vigor or heterosis [15]. To achieve this, inbred parental lines are first developed, and these are used to perform a uni-directional cross at the final stage to deliver the heterotic F_1_ product. However, currently commercial exploitation of hybrid vigor is limited to a relatively small number of crops despite the evidence that heterosis above high parent exists within many species. The floral biology of the crop, the nature of pollination and natural out-crossing rate in crops plays a significant role in heterosis. Development of hybrid cultivars in some of the self-pollinated crops such as lentil has several challenges, e.g., inability to produce enough seeds per cross, possibility of outcrossing, absence of male sterile lines all of which makes it difficult to commercialise hybrids on an economic scale [16]. Benefits of heterosis can only be harnessed if F_1_ seed production can be scaled up using innovative approaches. However, there are some hybrid cultivars in self-pollinating crops such as, egg plant, tomato, and pepper, with 30–60% hybrid yield advantage which is achieved due to perfect size of flower, many seeds per cross and with natural out-crossing rate close to null [17].

Heterotic F_1_ hybrid can be achieved by F_1_ hybrid-based breeding systems, such as artificial or chemical emasculation; cytoplasmic or nuclear male sterility [18]. In the absence of highly efficient pollination control technology, a new alternative pathway to deliver heterosis has been proposed via hybrid mimics where the first level of hybrid conventional selection from the pure breeding lines are held and selected for at every generation until the desired characteristics of F_1_ hybrid are stabilised [19]. Heterosis via F_1_ hybrids has been realised in both self and cross pollinated crops such as rice, wheat, *Brassica napus*, sorghum, sunflower, rye, onion, tomato, peppers, and barley [20–23] with up to 30% yield gains and its utilization has contributed greatly to global crop production with high level of hybrid adoption [20]. The use of hybrid maize since its inception has significantly contributed to the yield gains realised, these production gains helped expand hybrid maize acreage from less than 10% to over 90% in Iowa, United States from 1935 to 1940 [24]. Heterosis is often reported as trait-specific, for instance, tomato hybrids were found to be highly heterotic for seed number per plant, fruit number and total yield but other phenotypes such as fruit weight and seed morphology displayed no heterosis [25]. Additionally, Flint-Garcia et al. [26] identified heterosis for yield in maize whilst flowering time was additive.

To explore the underlying genetic principles of heterosis, several models have been proposed [27–30]. Heterosis through dominance occurs when the dominant alleles from one parent suppress the inferior recessive alleles from the other parent. With this model, it is theoretically possible to eliminate all deleterious alleles and/or accumulate all desired alleles to create an inbred line performing similar to the F_1_ hybrid. The overdominance hypothesis details the superior performance of the hybrid due to the heterozygosity that delivers advantages over and above homozygous state. The third model is epistasis which explains the scenario where two or more non-allelic genes derived from the parental lines interact to generate heterosis. Several studies have been undertaken to understand the genetic mechanism of heterosis, however there is still no single genetic model to accurately predict the range and quantify the level of heterosis [21]. It is possible that alleles accumulate or interact with contributions from different models to explain the molecular mechanism of heterosis [20].

Understanding and quantifying heterosis in lentils will help explore the opportunities and potential gains that hybrid breeding could deliver to the agriculture industry. However, there are currently no reliable means to deliver heterotic F_1_ cultivars in a commercial ready system in lentils. F_1_ hybrid-based breeding systems, such as artificial or chemical emasculation; cytoplasmic or nuclear male sterility can be explored in lentils. Another potential solution could be hybrid mimics as a simpler way to deliver the benefit of heterosis without the need for complex seed multiplication and production systems. Hybrid mimics have been reported in wheat, peas, tomato, and tobacco [31–33]. Hybrid mimics have been exemplified in Arabidopsis [19] however, they have not been commercially exploited so far. Therefore, it is worthwhile evaluating the potential of hybrid mimics in lentils to explore the challenges associated with their commercial use.

The objectives of this study are to identify and quantify heterosis by phenotypic evaluation and selection for various developmental and yield related traits in lentil hybrids through multiple generations. To evaluate the range of heterosis in lentils, the percent yield gains were compared to other crops to model and inform their potential benefit for the lentil production industry. To explore if heterosis is trait specific and to detail highly heterotic traits in a more accurate way, correlations among measured traits were explored. Genetic diversity of a set of parental lines was performed to examine correlations between genetic divergence and heterosis. Furthermore, the study evaluated and considered the heterosis results observed to understand the potential of delivery via hybrid mimics. Recommendations on future potential hybrid breeding schemes in lentil breeding were made.

## Material and methods

### Plant material and generation advance

**Fig 1:**
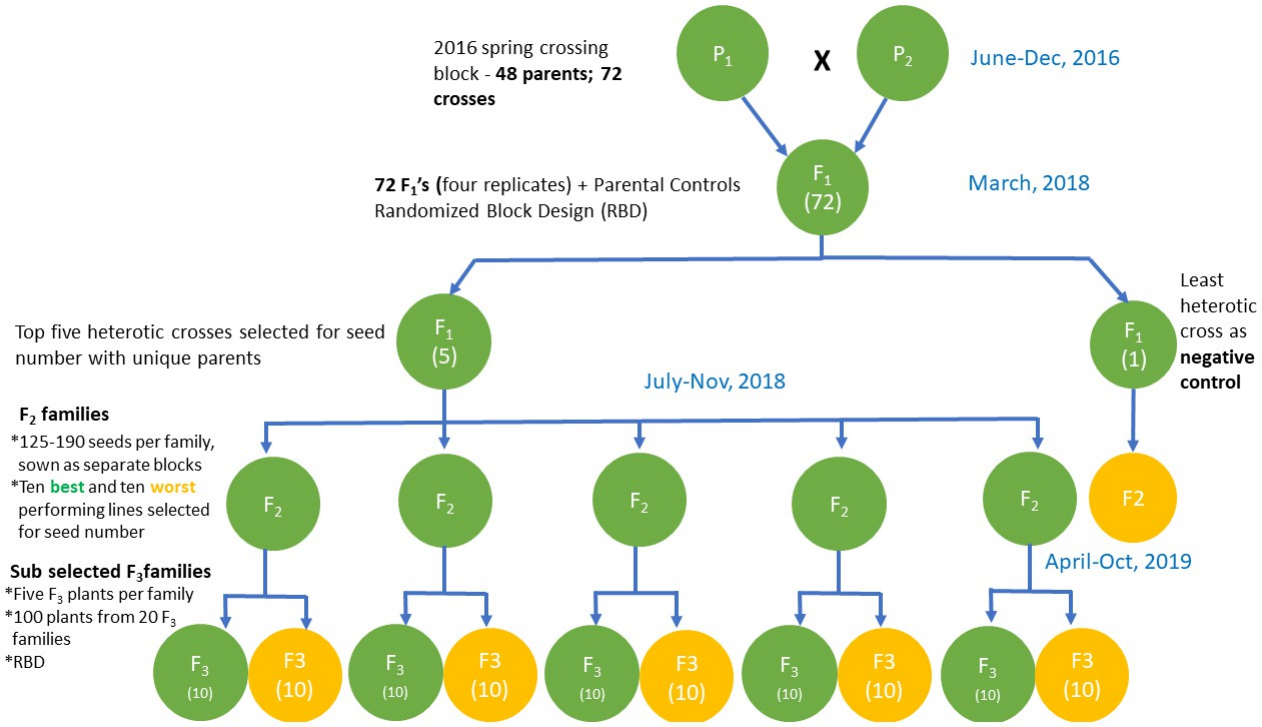
A summary of the generation advance in lentils, covering F_1_ to F_3_. The plants at each generation were evaluated for seed number, seed weight, biomass fresh weight and plant height.

A total of 47 lentil genotypes (S1 Table) selected on the basis of genetic diversity, yield and its component traits, such as seed number, plant height and biomass fresh weight (S1 Table) were cross hybridized using multiple parental combinations listed in S2 Table by hand pollination, over the spring of 2016 at The Grains Innovation Park, Horsham, Victoria, Australia and progressed to develop subsequent generations in a glasshouse under controlled environmental conditions (Fig 1). A total of 72 F_1_ hybrids as well as parent genotypes were initially evaluated in a randomized complete block design consisting of 4 replicates for seed number, yield, biomass fresh weight, and plant height with six crosses being taken forward to the next generation. The F_1_ crosses were numbered in descending order based on levels of heterosis. The top five F_1_ better parent heterotic crosses in terms of seed number were chosen and a filter on genetic diversity was also imposed to ensure that multiple genetics were selected. Along with the top five performing crosses, a negative heterosis control cross-72 was also selected. At the F_2_ generation, 125–190 seeds per family were sown as separate blocks (Fig 1) and the populations were not intermixed across blocks. While designing the glasshouse trial, replicated parental controls were present in every block to evaluate environmental variance. Within the block structure, six replicates of each parent were distributed within the relevant population for all generation trials. For every F_2_ family the ten best performing lines and ten worst performing lines in terms of seed number were taken forward as sub selected F_3_ families (20 in total per F_1_ cross) for evaluation. A total of five F_3_ plants per family (total of 100 plants from 20 F_3_ families that relate to each F_1_ cross) were screened in a randomised block trial (Fig 1).

### Growth conditions

All plants were grown under controlled environment conditions using standard potting mix (1.3 L) in 140 mm plastic pots at 22 ± 2°C with a photoperiod of 16/8-h (light/dark). Both F_1_ and F_3_ generations were evaluated using a randomised complete block design and F_2_ was evaluated with blocking structure around families with randomised parents in every block. A minimum of four replicates were evaluated for each parent at all three generations in every cross to evaluate environmental variation.

### Phenotypic evaluation

A number of yield related traits were measured in each experiment (F_1_, F_2_, F_3_ generational assessment) to quantify heterosis, where each individual was assessed for seed number, seed weight, biomass fresh weight and plant height at time of harvest. Each plant was harvested by taking all the above ground biomass, packing the biomass in a pre-measured paper bag, and weighed for biomass fresh weight on a balance (Thermoline Scientific Precision Balance, WLC 6/A2, d = 0.1g; New South Wales, Australia). Subsequently, these plants were oven-dried at 37°C for 48–72 h and threshed for seeds. Seeds were collected from each individual plant and the number of seeds generated was counted using an automatic seed counter (Data Count, JR; Data Technologies, Tzora, Israel) as well as generating a total yield value in terms of total seed weight per plant. In addition, a hundred seed weight per plant value was calculated by dividing the total seed weight by the number of seeds and then multiplying by a hundred. The data was evaluated for mean, and standard error using ASREML (v 4.1.0) [34].

### Phenotypic data analysis

For the F_1_ phenotypic data, a spatial analysis was performed, and predicted mean values were calculated for seed number, yield, biomass fresh weight, and plant height using ASREML [34]. In addition, for each trait under study one-hundred numeric values were simulated using the predicted mean values along with standard errors assuming a normal distribution.

For the F_2_ and F_3_ generation evaluation, the data was analysed on a single plant basis since it was not possible to have genetic replicates of the population. However, mean values of the replicated parents along with their standard errors have been considered to understand environmental variance in the glasshouse trial.

Correlation coefficients between plant height, biomass fresh weight, seed number, and yield per plant were determined using R software (v4.0.0).

The percentage of heterosis was analysed in F_1_, F_2_ and F_3_ as better parent heterosis (BPH), which was computed BPH = (F_1_-BP)/BP × 100, where BP referred to the better-parent value.

Multiple Linear Regression analysis was performed in F_2_ and F_3_ generations using library Tidyverse in R software (v4.0.0). A single multivariate regression model was created to predict yield based on four predictor variables, such as, biomass fresh weight, plant height, seed number and hundred grain weight.

### Genotypic data of parental varieties

Leaf tissues from multiple nodes were collected from 4 weeks-old plants and were frozen immediately in liquid nitrogen and stored at −80 °C until required. Total RNA (tRNA) was extracted using RNeasy^®^ Plant Mini Kit (QIAGEN, Hilden, Germany) following manufacturer’s instructions. RNA-Seq libraries with an approximate insert size of 350 bp were prepared using Sure Select Strand Specific RNA library prep kit and evaluated using the Tape Station 2200 platform with HSD1000 Screen Tape System (Agilent Technologies, Santa Clara, CA, USA) according to the manufacturer’s protocols. Equal mass of each sequencing library with a unique barcode was combined to create a single pooled sample for sequencing. All reads were pair-end sequenced using the HiSeq 3000 and MiSeq platforms (Illumina Inc., San Diego, CA, USA). Raw data has been deposited to NCBI under accession number GSE184819 (Advances in lentil production through heterosis: evaluating generations and breeding systems (lentil)); Data Type: transcriptome or gene expression).

Following Fastq data generation, the raw sequence reads were filtered using a custom perl script [35] to remove adaptor sequences along with reads and bases of low quality (Q ≤ 30). Reads with three consecutive unassigned nucleotides (N) were also trimmed and finally any reads shorter than 50 bp in length were removed from the final set. The remaining high-quality trimmed sequence reads were aligned to the lentil reference transcriptome [36] using BWA-MEM [37]. The number of properly paired reads were obtained using the SAMtools flagstat option and the mapping reads were obtained [38]. Variant calling was performed using SAMtools (version-1.5) [38]. The final VCF output was then filtered using VCFtools [9] based on the following parameters: depth (DP ≥ 5), maximum allelic frequency (MAF = 0.1), maximum missing data (20%), and base quality (Q30) with a predefined SNP list [39].

Genetic diversity analysis was performed using the abovementioned filtered SNP data from all accessions. Genetic distances for each lentil accession were calculated using Nei’s method within the StAMPP package [40]. A phylogenetic tree was constructed using the unweighted neighbour-joining (NJ) method, as implemented in the DARwin-6.0.17 software [41].

## Results and discussion

A total of 72 biparental crosses between varying lentil genotypes were established (S2 Table). Of the resultant F_1_’s, 43 crosses exhibited positive heterosis based on seed number. Overall, the range of heterosis observed varied between -59% to 62%, with 18 out of 72 crosses exhibiting greater than 20% heterosis (S2 Table). The crosses were numbered from one to seventy-two in descending order of heterosis at the F_1_ generation for the trait of seed number. The F_1_ data suggested the presence of heterosis in lentils and identified some of the best performing crosses for detailed analysis of the phenomenon.

### Patterns of heterosis for seed number, yield, and biomass fresh weight

**Fig 2:**
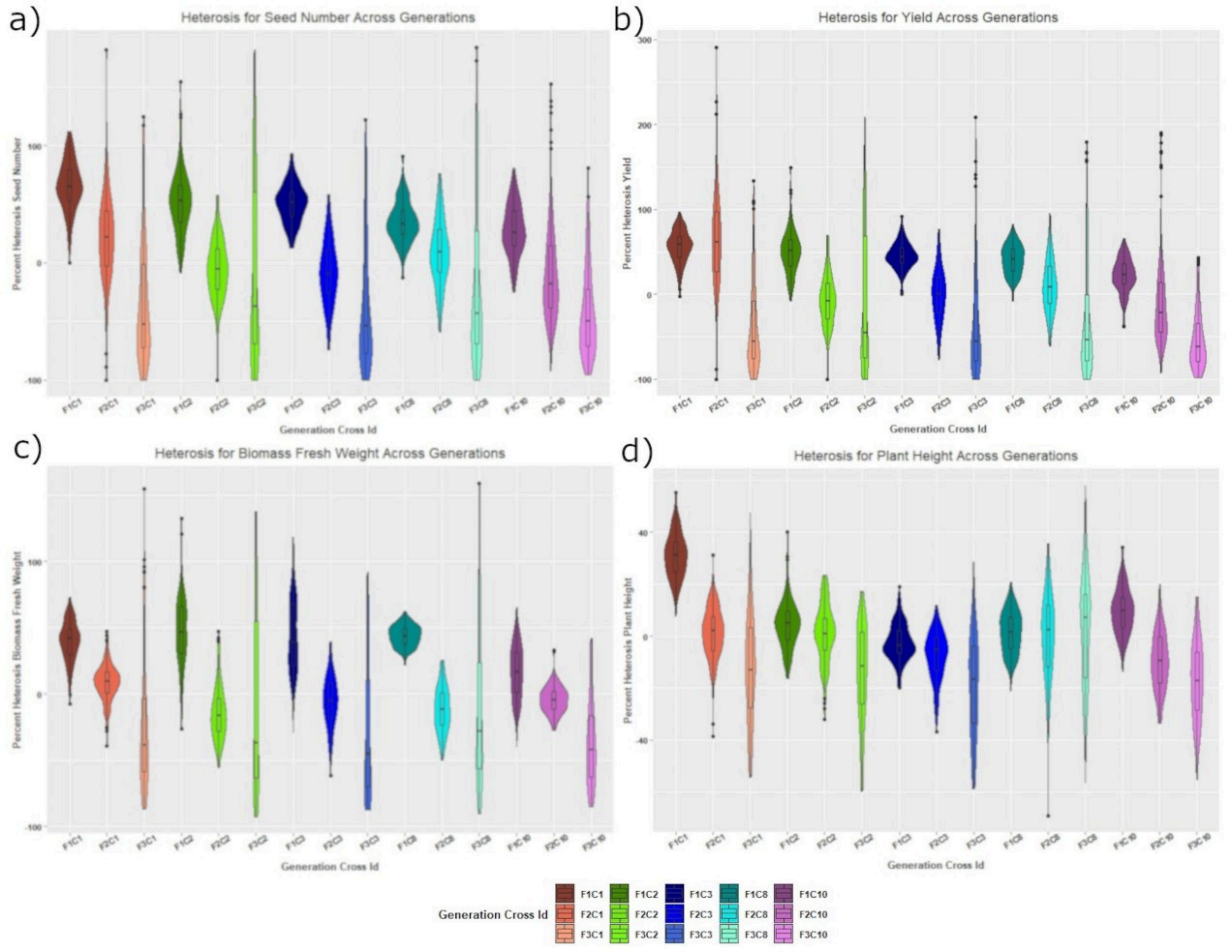
F_1_, F_2_ and F_3_ hybrids from selected crosses (Cross-1, 2, 3, 8 and 10) evaluated for better parent heterosis in terms of a) seed number b) seed weight c) biomass fresh weight and d) plant height.

Heterosis for seed number, yield, and biomass fresh weight of the five positive heterotic crosses performed in a similar way and exhibited maximal positive gains at the F_1_ generation with an overall decline in the population mean in subsequent generations. However, despite the decline in heterosis, the distribution of the individuals within the generation broadened (Fig 2a–2c).

Heterosis for seed number, yield, and biomass fresh weight at the F_1_ generation was quantified in the range of 31%–62%, 26%–56% and 18%–50%, respectively across the five selected crosses with a global average of 31±1% (Table 1) quantifying heterosis from some of the best performing crosses for the detailed analysis of the phenomenon. Heterosis for seed number is one of the important traits affecting seed yield. Nearly 11% heterosis was observed for the number of seeds per silique in F_1_ *B*.*napus* hybrids [42]. Similar to this study, 10%–30% heterosis for yield has been reported in wheat, maize, rice, pea, soybean, and *B*. *napus* in F_1_ heterozygous generation [20, 43]. Dinkins et al. [44] reported 12% heterosis for biomass in F_1_ hybrids of soybean and in comparison, 18%–50% heterosis for biomass was observed in the current study. Better parent heterosis for yield (25%) has also been recorded in commercial F_1_ hybrid cultivars of Chinese cabbage [45].

**Table 1:** Mean value of percent heterosis of selected crosses (cross-1, 2, 3, 8 and 10) along with negative control (cross-72*) for seed number (SN), yield, biomass fresh weight (BFW) and plant height (Ht) at F_1_, F_2_ and F_3_. The table includes Maximal (Max) and Minimal (Min) percent heterosis values at F_2_ and F_3_ along with parents represented as Mean±S.E.

		Cross-1	Cross-2	Cross-3	Cross-8	Cross-10	Cross-72*
**F**_**1**_	**SN**	61.9	56.78	51.63	35.26	30.69	-59.21
	**Yield**	56.2	54.79	45.68	41.69	25.72	-34.64
	**BFW**	39.94	49.82	46.26	44.2	17.74	-24.11
	**Ht**	31.23	6.21	-2.68	1.86	9.88	-17.07
**F**_**2**_ **-SN**	**Mean**	20.45	-4.99	-8.32	8.79	-7.72	-46.35
	**Max**	181.45	57.64	58.44	76.35	152.47	-11.43
	**Min**	-88.71	-58.95	-74.63	-58.92	-92.4	-91.43
	**P-1**	124±8.55	121.8±6.74	145.8±6.53	99.8±13.50	131.5±14.13	105±24.29
	**P-2**	85.33±15.61	112±21.52	114.4±14.17	92.6±14.10	127.67±32.91	65±5.59
**F**_**2**_ **-Yield**	**Mean**	63.47	-6.81	-0.03	9.99	-8.99	29.31
	**Max**	291.18	69.85	76.95	95.65	190.32	116.67
	**Min**	-88.24	-59.92	-77.27	-61.35	-92.74	-80.55
	**P-1**	3.4±0.31	5.24±0.41	6.16±0.37	4.14±0.71	3.7±0.40	3.6±0.89
	**P-2**	3.03±0.62	4.64±0.92	5.48±0.84	3.74±0.63	4.13±1.32	2.76±0.31
**F**_**2**_ **-BFW**	**Mean**	8.52	-13.91	-6.09	-11.67	-4.51	8.31
	**Max**	46.88	47.26	39.24	25.61	33.06	52.99
	**Min**	-39.18	-55.71	-61.37	-49.84	-27.69	-66.79
	**P-1**	29.23±2.07	12.78±0.58	15.92±0.95	15.28±1.27	34.6±0.79	13.19±1.88
	**P-2**	32.88±1.70	12.72±1.12	15.44±1.78	16.41±1.23	37.2±1.46	9.57±0.49
**F**_**2**_ **-Ht**	**Mean**	1.23	1.02	-7.92	-0.15	-9.66	-3.82
	**Max**	31.34	23.46	11.99	35.71	19.78	18.47
	**Min**	-38.58	-32.1	-36.91	-69.05	-33.46	-33.79
	**P-1**	97.5±4.13	48.6±1.51	63.4±1.54	55±1.47	73.33±3.38	57.4±3.54
	**P-2**	105.83±2.34	48.2±1.04	56.6±2.85	55.6±2.97	90.17±3.14	44.4±1.31
**F**_**3**_ **-SN**	**Mean**	-34.44	-4.67	-34.21	-19.89	-41.91	NA
	**Max**	124.66	181.99	121.71	184.06	81.04	NA
	**Min**	-97.81	-97.96	-93.14	-95.94	-95.56	NA
	**P-1**	78.25±14.25	152.75±37.78	175±43.88	90±29.45	160.5±30.63	NA
	**P-2**	273.75±84.15	195.75±48.52	131.75±30.54	172.5±41.57	292.75±34.57	NA
**F**_**3**_ **-Yield**	**Mean**	-35.62	-4.78	-30.08	-27.57	-51.93	NA
	**Max**	133.67	209.78	208.66	179.73	43.33	NA
	**Min**	-98.38	-98.91	-93.71	-97.3	-98.5	NA
	**P-1**	2.43±0.43	7.25±2.03	6.35±1.78	3.48±1.22	5.63±1.27	NA
	**P-2**	12.33±4.12	9.2±2.70	4.78±1.28	7.4±2.23	13.33±1.96	NA
**F**_**3**_ **-BFW**	**Mean**	-23.13	-6.13	-27.16	-11.23	-36.36	NA
	**Max**	154.68	137.99	91.85	159.19	42.02	NA
	**Min**	-87.01	-92.63	-87.38	-90.42	-84.98	NA
	**P-1**	8.08±1.51	16.01±3.14	21.79±5.09	18.35±6.30	15.51±1.05	NA
	**P-2**	26.56±7.99	22.38±4.15	12±2.71	22.98±3.29	27.64±2.74	NA
**F**_**3**_ **-Ht**	**Mean**	-11.78	-14.44	-18.99	2.82	-17.52	NA
	**Max**	47.6	17.26	28.41	58.06	15.25	NA
	**Min**	-54.24	-59.61	-58.67	-56.45	-55.25	NA
	**P-1**	42.5±4.07	72.75±4.93	67.75±6.61	49.75±4.22	58±1.41	NA
	**P-2**	67.75±5.76	76.75±4.32	43.25±5.21	62±2.48	73.75±3.38	NA

Heterosis was further investigated at the F_2_ generation (Fig 2a–2c) with the mean heterosis values for the traits of seed number, yield and biomass fresh weight being -8% to 20%, -9% to 63%, -14% to 9%, respectively across five crosses (Table 1). The negative heterosis control cross at the F_2_ stage had a migration of the mean value towards the zero heterosis level, clearly following the same trend but demonstrating a loss of negative heterosis rather than a loss of positive heterosis. However, this decrease in heterosis is in concordance with the classical theory of heterosis [46, 47]. Similarly, Sarawat et al. [48] observed up to 50% reduction in heterosis at F_2_ as compared to the F_1_ for grain yield and total dry matter in peas. Wang et al. [19] also noticed that yield advantage of the F_1_ is lost in the F_2_ and subsequent generations in Arabidopsis. Another study by Burton and Brownie [49] reported that the yield advantage declined from 16% to 5% as the hybrids progressed from F_1_ to F_2_ in soybean. There are other reports for significant decline in heterosis with the subsequent generations in soybean [43, 50]. Scheffler et al. [51] also reported in maize the F_2_ generation averaged 32% less grain yield than the F_1_ generation.

Although, the mean values of heterosis decreased at F_2_ as compared to F_1_, the spread of values significantly increased across the populations so there were a small proportion of individual genotypes that displayed positive heterosis. For instance, the maximal heterotic values for seed number varied from 58% to 181% across five crosses at F_2_. These rare extreme genotypes delivering positive heterosis from the population at F_2_ had heterosis values higher than the mean F_1_ value with an exception of crosses-2 and 3 (Table 1). These rare extreme genotypes account, at the F_2_ generation for 2% to 20% of individuals for the trait of seed number across all five best performing crosses (S3 Table). A similar observation has been reported by Sarawat et al. [32, 48] where some of the F_2_ populations maintained the high yield levels of the corresponding F_1_ hybrids in pea. Singh et al. [52] also observed the outliers at F_2_ in pea.

To understand the trend of heterosis at further generations, F_2_ plants that had maximal and minimal values for seed number, were evaluated as the sub selected F_3_ generation (Fig 2a–2c). The mean heterosis values for seed number, yield and biomass fresh weight varied from -42% to -5%, -52% to -5% and -37% to -6% respectively across five crosses (Table 1), representing a decrease in the mean value of heterosis for all traits in comparison to both the F_1_ and F_2_ generations. This generation advance did not perform as expected, with the offspring from both tails of the F_2_ distribution performing comparably. The sub-selected F_3_ generation also was the first point where the mean values for all traits in all crosses recorded negative values. However, despite the mean negative values of heterosis at this stage, there were again a small number of individuals, a comparable proportion of the population as was identified at the F_2_ generation (5% to 29%), with phenotypes at the extreme of the distribution that performed better than the F_1_ generation mean value (Table 1 and S3 Table). This variation should be expected as a high number of loci at F_2_ would still be segregating. The evaluation of environmental variance of all the genotypes in the trial was still not possible with segregating individuals unable to be replicated as previously discussed. However, the environmental variation as assessed by the parental genotypes provides some quantification of the variance that was experienced in the trial, and reassuringly, the extreme values from the population were still positively heterotic even considering the environmental variance as estimated through the parental replicates. Fischer and Rebetzke [53] also discussed similar issues relating to early generation selection for yield in conventional breeding systems in self-pollinated crops, where allelic segregation and recombination in early generations (F_2_-F_4_) leads to unreliable selection before homozygosity is attained. As a result, future studies would be suggested to be conducted from the entire F_2_ derived F_3_ families rather than making selections and taking the tails of the distribution. Alternatively, it might be suggested to ignore the F_2_ generation evaluation entirely and simply perform a seed multiplication to advance generations and useF_2:3_ families to retrospectively calculate F_2_ genotype values. This would allow a form of within family replication. Alternatively modelling of rates of selection, recombination and inbreeding could indicate the most efficient strategy to advance either the entire populations or sub selected fractions to achieve advanced populations that would still have small proportions of the extreme heterotic individuals.

The identification of individuals at sequential generations that outperform the F_1_ generation supports the concept that the hybrid mimic breeding strategy could be deployed for lentil varietal development. This approach utilizes recurrent selection for the critical hybrid vigor trait to advance generations while stabilising and multiplying the population to retain heterosis in pure breeding lines [15, 19]. Researchers have been able to select hybrid mimic like plants in a number of species, for instance Busch et al. [31] reported that the pure breeding F_5_ lines derived from hybrid plants performed equivalent to F_1_ hybrid in wheat. Similar findings have been reported in pea and tomato where researchers have been able to develop stable F_5_-F_6_ lines with the same characteristics as the parental F_1_ hybrids [32, 33]. Sarawat et al. [32] reported equivalent performance of F_5_ lines in pea as compared to F_1_ hybrids (out yielded the best parent by up to 11%). Similar findings have been reported in pea, tomato, and tobacco where researchers have been able to develop stable F_5_-F_6_ lines with the same characteristics as the parental F_1_ hybrids [32, 33, 54]. Although there are now several reports of the development of hybrid mimics, it is still challenging to extend the hybrid advantage beyond F_1_ and as discussed, the method of generation advance needs careful work, consideration, and experimentation. Sarawat et al. [32] reported a similar percentage of the population (2–15%) compared to this study at F_5_ performed better than the F_1_ mean. The limited scale of valuable lines and attrition in the multiplication process does present additional logistical challenges on a commercial scale for hybrid mimics and necessitates the screening of a large number of lines and reliable germplasm to work with. It is likely that due to the aforementioned complexities, hybrid mimics have not been more widely commercially exploited so far.

It is understood that there are some challenges associated with the development of hybrid mimics. An alternative way to utilise heterosis is through wide hybridisation heterosis using pollination control mechanisms. Different pollination control systems have been used to breed for heterosis. In some crops such as rice, maize, wheat, *B*. *napus*, and sunflower, cytoplasmic or nuclear encoded male sterility has been explored [18]. Future research activities could focus on the identification and development of male sterile lines in lentil, through natural or induced mutations. There are reports in the literature of induced mutants in lentils for male sterility already [55] and further research on this is likely to prove fruitful.

### Patterns of heterosis for plant height

In comparison to other traits in the study, the F_1_ hybrids exhibited a subtly different pattern of heterosis for plant height. As compared to other traits, the level of heterosis was significantly lower for plant height except for cross-1 (Fig 2d). Heterosis for plant height varied from -3 to 32% at F_1_, -10 to 1% at F_2_ and -19 to 3% at sub-selected F_3_ (Table 1). The populations exhibited overlapping distributions between F_1_ and F_2_ generations, however, a wider distribution was noticed at the sub-selected F_3_. Although extreme phenotypes were observed for plant height, the extreme performance was lower than that observed for seed number, yield and/or biomass fresh weight. The highest value from the population for heterosis for plant height was 36% and 58% at the F_2_ and sub selected F_3_ generation respectively (Table 1). There have been previous studies, which clearly demonstrate the trait specific nature of heterosis [25, 56, 57]. In *B*. *napus* and soybean, positive heterosis was seen for yield related traits (1000 seed weight and seed yield) but not for plant height [56, 57]. van Hulten et al. [58] reported that the genetic mechanisms underlying heterosis were highly trait specific and were largely dependent on the genetic background. In agreement with the aforementioned studies, the trait specific nature of heterosis is confirmed for lentil and further, it is necessary to identify and establish the correlations between production traits on a species and potentially genotype basis. However, the exploration of heterosis in other agronomically important traits, such as flowering time, maturity time and plant architecture, could be of value to lentil breeders if it can be shown how these traits relate to yield.

### Correlation studies across generations

A correlation analysis was undertaken to investigate the consistency of heterotic patterns between the different crosses (S4 Table). Multiple linear regression analysis has also been used to explore the relationship between independent or predictor variables (S5 Table). Generally, positive correlations between yield traits are beneficial in improving productivity through conventional breeding. In the current study, yield was significantly and positively correlated with seed number, biomass fresh weight, plant height and 100 grain weight in all six crosses across the F_1_, F_2_ and sub selected F_3_ populations (r = 0.23–0.99) except for cross-2 where yield was not significantly correlated to 100 grain weight at F_1_. Also, cross-1 showed no significant correlation between yield and plant height at F_2_. The degree of correlation was evaluated across the generations in all the crosses under investigation and it was observed that yield correlated to plant height (r = 0.23–0.99), biomass fresh weight (r = 0.37–0.98), seed number (r = 0.32–0.98), and 100 grain weight (r = 0.26–0.66). Multiple regression analysis also confirmed that yield per plant at F_1_, F_2_ and sub selected F_3_ generation could be predicted by hundred grain weight (coefficient = 0.061–2.10) and seed number (coefficient = 0.01–0.06) within each generation in all the populations under study (multiple R-squared values > 0.91, S5 Table). Broadly, the correlation study suggested that plants with greater biomass fresh weight, yielded a greater number of seeds with higher yield per plant. Parallel to the current study where yield correlated with seed number and biomass fresh weight in all the six crosses under study, similar observations have been reported in *B*. *napus* where seed yield correlated significantly with nine yield related traits (including seed number, biomass yield, pod number, and plant height) for both trait performance and mid parent heterosis [59]. Significant correlations between yield related traits have been observed in a number of other crops such as tomato, wheat, maize, and sunflower [25, 60–63]. There are reports where high heritability values have been recorded in lentil for grain weight (h^2^ = 0.87) but moderate heritability has been observed for yield (h^2^ = 0.5; [64, 65]. This indicates that grain weight experiences less genotype x environment interactions, whilst yield can be heavily affected by the environment. Therefore, it is important to understand the correlations between genetically controlled traits and traits impacted by environmental variance. Further, correlation studies will be useful for the development of hybrid mimics and evaluation of germplasm for generation advance. Efforts are also required to extend the work from this controlled environment study to a field setting.

### Genetic diversity analysis

**Fig 3:**
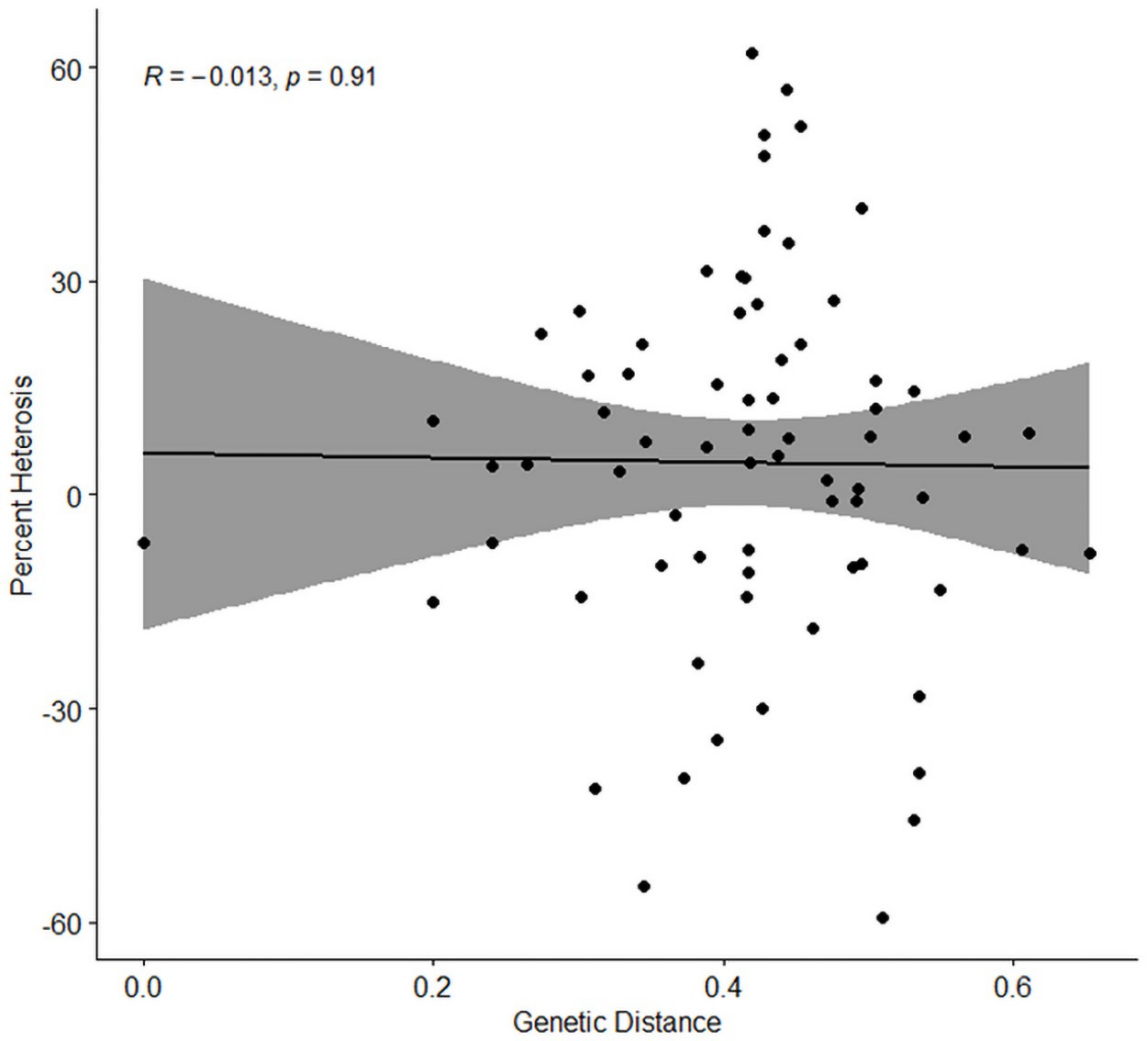
Correlation between genetic distance (nei’s genetic distance) of the selected parents and percent heterosis for seed number at F_1_.

**Fig 4:**
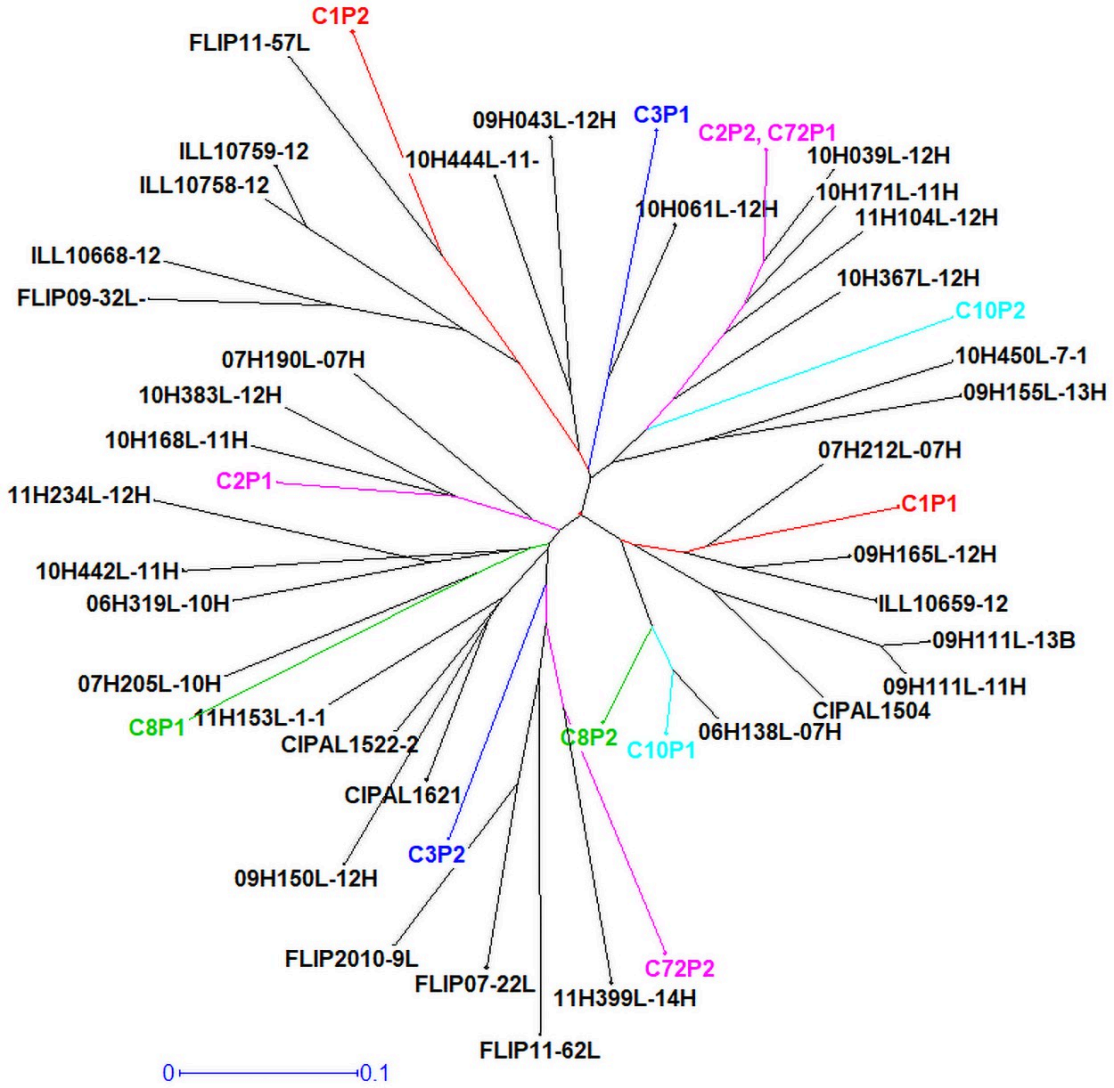
Phylogenetic tree of lentil parents used for creating crosses under heterosis study. The parents of the selected crosses used in the study have been colour coded and abbreviated for ease of visualisation, e.g C1P1 = Cross-1; Parent-1.

In an attempt to evaluate and predict heterosis and heterotic combinations, a genetic diversity analysis was performed, and the full set of parents used to create the 72 initial F_1_ crosses were evaluated for their genetic distances and relationships (Fig 3). The genetic distance matrix along with a phylogenetic tree revealed that genetic distance between all the parents of F_1_ hybrids was in the range of 0.049–0.68. Broadly, looking at the top ten heterotic crosses in the phylogenetic tree, they are not most genetically consistent and do not provide a clear picture of heterosis, however, they sit on different clades along with some non-heterotic crosses (S1 Fig). Narrowing the results to the selected six crosses, the average genetic distance between parent 1 and parent 2 of a particular F_1_ hybrid was 0.45 and across other crosses was not less than 0.23. This gives an initial set of boundary parameters for further evaluation to test genotypes with a degree of diversity for heterosis. There was a commonality of genetics between parents of cross-2 and cross-72, apart from being genetically distant (genetic distance between parents of cross-2 and cross-72 was 0.36, 0.51, respectively) they did not exhibit heterosis (Table 2; Fig 4). From this analysis it is evident that genetic distance alone does not predict heterosis, although all the crosses had distance values greater than 0.23, which is an indicator that can be derived from this approach (Table 2). The maximal heterotic crosses also clearly failed to define specific groupings or pools. More crosses are needed to dissect the relationships and provide finer resolution over the nature of heterosis in lentils. There have been reports for both positive (within a range of divergent parental lines) and negative (when the genetic distance was extremely high) correlation between genetic distance and heterosis [66]. A positive correlation between genetic distance and heterosis has been discussed by Birchler et al. [27]. However, there have been several studies where genetic distance and heterosis are not always positively correlated [67–69]. Van Hulten et al. [58] observed no correlation between heterosis levels and the genetic distance between parental lines. Fujimoto et al. [20] suggested that F_1_ hybrids between genetically distant parents would not necessarily produce heterotic F_1_ hybrids, only specific combinations of parental lines exhibit heterosis relative to the parental lines which correlates well to the current study. Maximal gains in performance are most likely to be realised if heterotic loci can be identified and selected for genomically from specific lentil plants.

**Table 2:** Genetic distance (Nei’s genetic distance) between the parents of the heterotic crosses under study.

	C1P1	C10P1	C10P2	C1P2	C72P2	C3P1	C3P2	C8P2	C2P2, C72P1	C2P1	C8P1
C1P1	0										
C10P1	0.27	0									
C10P2	0.43	0.41	0								
C1P2	0.42	0.52	0.56	0							
C72P2	0.50	0.40	0.58	0.48	0						
C3P1	0.47	0.37	0.40	0.55	0.41	0					
C3P2	0.47	0.34	0.46	0.50	0.39	0.45	0				
C8P2	0.23	0.12	0.43	0.48	0.40	0.36	0.33	0			
C2P2, C72P1	0.48	0.43	0.38	0.60	0.51	0.33	0.43	0.43	0		
C2P1	0.39	0.26	0.47	0.54	0.51	0.38	0.27	0.29	0.36	0	
C8P1	0.46	0.44	0.41	0.50	0.60	0.57	0.45	0.44	0.43	0.45	0

## Conclusion

Heterosis does exist in lentils and up to 62% heterosis being identified at F_1_ in a controlled environment. However, the yield advantage diminishes with the successive generations, but despite of the reduction, the range of heterosis spreads broadly and some individual high performing genotypes were identified at F_2_ and sub selected F_3_ generations. Heterosis was evaluated for yield related agronomically important traits and it was noticed that yield correlated with seed number, biomass fresh weight, plant height and 100 grain weight. Further, to explore the reasons underlying heterosis, genetic diversity analysis of all the F_1_ parents was performed and the study failed to identify a correlation between genetic distance and divergent parents with heterotic F_1_’s. Thus, the study can be further utilised to explore ways to achieve potential yield gains from heterosis either by F_1_ hybrid-based breeding systems and/or via modelling through computational simulation of different breeding programmes and recombination patterns to inform the best way to develop hybrid mimics. Despite the potential challenges, the potential yield gains in lentil from heterosis has the ability to transform the industry and warrants further efforts to realise the benefits.

## Supporting information

S1 Fig Phylogenetic tree describing the position of top ten heterotic crosses (highlighted in coloured boxes) along with other less heterotic and non-heterotic crosses. (TIF)

S1 Table Mean value for plant height, seed number, biomass fresh weight and yield of 47 selected genotypes used for producing heterotic F1’s. (XLSX)

S2 Table Percent heterosis of F1 hybrids for seed number, yield, biomass fresh weight, and plant height. (XLSX)

S3 Table Percentage of individuals performing better at F1, F2 and F3. (XLSX)

S4 Table Correlation coefficients between growth and yield related parameters in different populations evaluated at F2 and F3 stage (*P≤0.5). (XLSX)

S5 Table Multiple linear regression analysis across generations in test populations. (XLSX)
