# Supplementary figures and images for: Advances in lentil production through heterosis: Evaluating generations and breeding systems
Source: PLoS One. 2022 Feb 18;17(2):e0262857. doi: 10.1371/journal.pone.0262857 (PMC8856536; doi:10.1371/journal.pone.0262857)

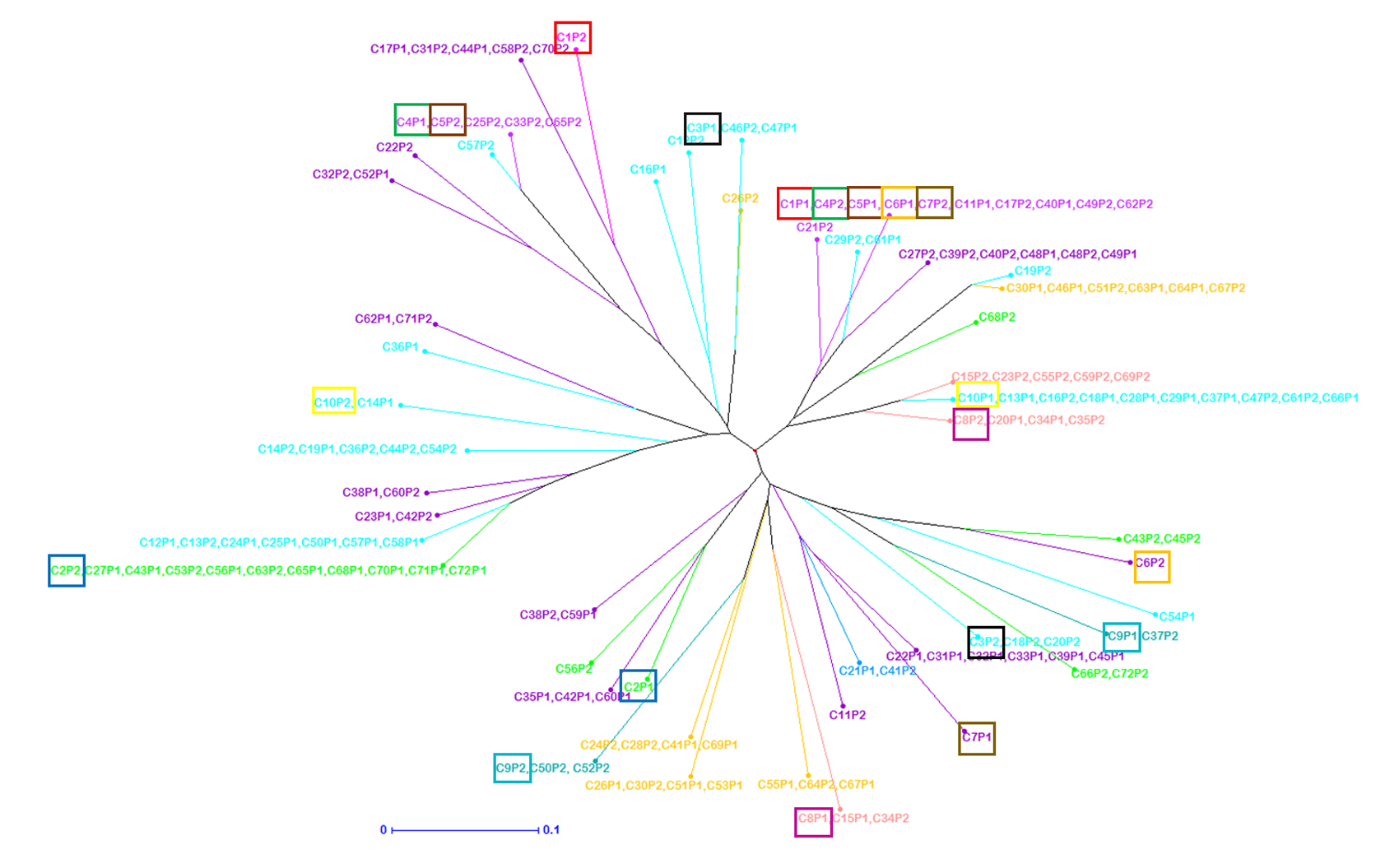

Supplement: S1 Fig — (TIF) [file pone.0262857.s001.tif]
